# Control of *Staphylococcus aureus* in dairy herds in a region with raw milk cheese production: farmers’ attitudes, knowledge, behaviour and belief in self-efficacy

**DOI:** 10.1186/s12917-018-1352-0

**Published:** 2018-02-13

**Authors:** Marie-Eve Cousin, Maria Christina Härdi-Landerer, Verena Völk, Michèle Bodmer

**Affiliations:** 1 Epidemiology, Biostatistics and Prevention Institute, University of Zürich, Hirschengraben 84, 8001 Zürich, Switzerland; 2 Institute of agricultural science, Animal Physiology, Swiss Federal Institute of Technology (ETH), Universitätsstrasse 2, 8092 Zürich, Switzerland; 3 Tierarztpraxis Dr. med. vet. Markus Nydegger, Scherlihalde 5, 3145 Niederscherli, Switzerland; 4 Clinic for Ruminants, Vetsuisse-Faculty, University of Bern, Bremgartenstrasse 109a, 3001 Bern, Switzerland

**Keywords:** *S. aureus*, Control-program, Attitude, Behaviour, Raw milk cheese quality

## Abstract

**Background:**

Contagious mastitis is an important disease in dairy cattle, and the causative agent *S. aureus* can also impair raw milk cheese quality. In a confined region in eastern Switzerland attitude, knowledge and behaviour towards *S. aureus* und *S. aureus* control was assessed in 90 dairy farmers with communal alpine pasturing including raw milk cheese production with the aid of a questionnaire.

**Results:**

Forty-three out of 90 questionnaires were returned (48% return rate). Farmers perceived reproductive problems as most important in their dairy herds followed by respiratory disease and diarrhoea in young stock. Most frequently stated as important motivating factors to participate in *S. aureus* control were “avoiding negative news about cheese quality in the press” followed by “I want to be proud of my somatic cell counts again”. Most frequently chosen and identified as important constraining factors were “I fear that the authorities dictate and the farmers are not heard” followed by “costs to control *S. aureus* are too high because of premature culling” and “I am afraid to be forced to cull genetically valuable cows”. Farmers with an experience of a *S. aureus* problem in their dairy herds had a significantly better knowledge about contagiosity and clinical manifestation of different *S. aureus* genotypes than farmers with no self-reported experience of a *S. aureus* problem. Veterinarians were indicated as the most important experts, farmers seek advice in case of mastitis and most farmers suggested subsidising bacteriological milk analysis as an incentive to motivate farmers towards *S. aureus* control.

**Conclusion:**

According to the results an improved knowledge transfer on *S. aureus* to dairy producers and an integrative approach to a *S. aureus* control program with subsidising milk analysis will be most promising to improve the *S. aureus* situation in this confinded region of eastern Switzerland. Veterinarians should cover a key role in consulting farmers during the control program.

**Electronic supplementary material:**

The online version of this article (10.1186/s12917-018-1352-0) contains supplementary material, which is available to authorized users.

## Background

Mastitis is one of the most important diseases in dairy herds [1]. This is especially true for herds selling milk for raw milk cheese production. For this particular niche production, milk needs to fulfill strict quality criteria to guarantee food safety in Switzerland [2].

In alpine parts of the country, communal alpine pasturing takes place during summer (May/June to September). The milk of lactating dairy cows is traditionally used for raw milk cheese production.

In the past 5 years, several cases of contamination of raw milk cheese with *S. aureus* enterotoxins were recorded in the eastern part of Switzerland and can be a threat for human health [3]. Staphylococcal enterotoxins are described as a cause of food poisoning leading to vomiting and diarrhoea in humans and cases of food poisoning by *S. aureus* enterotoxins deriving from dairy products were reviewed [4]. A recent report describes an outbreak of *S. aureus* enterotoxin poisioning due to raw milk cheese consumption in a boarding school [5]. In the year 2016, 4 confirmed cases were reportet to the Federal office for Public Health Switzerland [6].

*S. aureus* genotype B (GTB) [7] was one of the main isolates to be identified as cheese contaminants [3]. There are a variety of factors triggering enterotoxin production of *S. aureus* very much depending on the type of the enterotoxin and on other circumstances. However, the density of *S. aureus* bacteria within the milk (> 10^5^ cfu/g) seems to be crucial to activate accessory gene regulators [8]. Therefore, if *S. aureus* enterotoxins are detected in cheese, we may assume that the raw milk used for cheese production was already heavily contaminated with *S. aureus,* in particular with GTB [3]. In a recent study [9], it was shown that communal alpine farming during summer is a risk for the rapid spread of a particular *S. aureus* strain within the herds leading to high counts of *S. aureus* GTB in the raw milk destined for cheese production.

Until now, no targeted intervention for *S. aureus* control during alpine pasturing was implemented in the affected region, although effective control programs including testing, following a strict milking order according to *S. aureus*-status and culling of non-curable animals were suggested earlier [10]. The need for control programs on herd level is utterly important, since cure rates during lactation are disappointing [11]. They depend on different factors of the affected cow [11], on treatment duration [12] and on distinct characteristics of *S. aureus,* among others internalization into mammary gland cells [13], and forming of biofilms [14].

Although farmers think that their knowledge on current prevention practices is sufficient [15], they very often lack compliance for implementation of control measures [16]. The extent, to which mastitis is perceived as a problem is significantly different between dairy producers [17, 18], and it is likely, that a group of farmers cannot agree on a specific threshold and the measures to be implemented if the threshold is overcome as long as the official regulations are met. The most important factors for motivation to improve mastitis management were shown to be: job satisfaction, overall situation of the farm, economic loss, animal health and welfare awareness, ease in meeting regulatory requirements, extra financial incentives by bonus payments based on bulk tank somatic cell counts (BTSCC), dairy product quality, image and recognition for “job well done” [19]. The relatively low importance of economical aspects for motivation might as well explain the not fully rational reaction of farmers towards economical consequences of controlling somatic cell counts [20]. A study conducted in the United Kingdom showed, that extrinsic factors (i.e. financial barriers) were not relevant to farmers lacking intent to control zoonotic disease, as long as their believe in self-efficacy and their normative beliefs of responsibility towards consumers were absent [21]. Dutch dairy farmers rated the effectiveness of penalty strategies higher than bonus strategies regarding improvement of the mastitis situation in their herds [19]. Most of the above mentioned studies were conducted in the framework of a dairy production regulated by quota. Swiss dairy production is characterized by a milk market without quota, different local niches of raw milk cheese production with high demands of quality and an elaborate governmental direct payment system to support ecological production. It was shown before, that particularly self-reported attitude and knowledge of farmers contributed to a decrease clinical mastitis [22, 23]. Therefore, the aim of the present study was to investigate potential causes for insufficient compliance with *S. aureus* control measures by 1) assessing attitude towards *S. aureus* control considering communal alpine pasturing during summer, 2) assessing knowledge of farmers on contagious mastitis caused by *S. aureus* and in particular GTB, 3) assessing behaviour in the context of mastitis management and *S. aureus* control and 4) assessing their believe in self-efficacy in a confined region with a niche production of raw milk cheese and high amount of governmental subsidies.

## Methods

### Participants

Ninety farmers in a confined alpine region in eastern Switzerland who sent their dairy cows to 9 distinct communal alpine farms during 90 days in summer were included in the survey. Because of the low number of eligible respondents no random selection of participants was performed.

### Questionnaire

In order to assess attitude, knowledge, behaviour and self-efficacy of the farmers a structured questionnaire was created.

The questionnaire was based on the insight of 4 semi-structured interviews conducted with volunteers among the farmers of the respective region and contained 1) items about demographic farm-data, 2) items on general aspects of bovine health including fertility, claw health and calf health 3) items on knowledge about mastitis in general and *S. aureus* mastitis in particular, 4) items on behaviour concerning management of mastitis cases and mastitis prevention, 5) items on attitude towards mastitis prevention and *S. aureus* control, 6) items on self-efficacy and 7) items about different aspects of desired support during a potential *S. aureus* control program. Questions about infectious disease were not included since Switzerland is officially free of IBR, TBC, EBL and BVD. Additionally, no questions about endoparasitic disease were included since there are regionally agreed regulations on deworming of all heifers for communal alpine pasturing.

The interviewees agreed on a written consent that the provided information is exclusively used for research and only published in an anonymised fashion. The repsondents to the written questionnaire were also informed, that data they provided was only published in an anonymised fashion.

Demographic information concerning the farms was collected as continuous data (milk yield, herd size) or as categorical data (e.g. housing system, surface). Variables were categorised before analysis; details about categories are given in Table 1. The full questionnaire translated into English is added as Additional file 1.

**Table 1:** List of variables used for assessing demographic farm data

Variable	Unit categories
Surface of farm	< 10 ha
	10-15 ha
	16-25 ha
	> 25 ha
Number of dairy cows on farm	< 10; 10-15; 16-25; > 25
Number of calves on farm	continouus
Number of heifers on farm	continouus
Expenditure of time for farming by the farm owner	100%
	< 50%
	> 50%
Total equivalents of manpower on farm	Only 1 person……%
	Additional person 1 ……%
	Additional person 2 …..%
	Additional person 3……%
Main income	Dairy
	Veal calves
	Beef
	Egg production
	Broilers
Special production label	None
	BIO Suisse (organic)
	IP Suisse (integrated production)
	Terra Suisse
	Naturaplan
Heifer rearing	Only own heifers on own farm
	Only own heifers at external rearing farm
	Own heifers and purchases at external rearing farm
	Own heifers and purchases at own farm
	Only purchases
Number of purchases previous 12 months	None
	Heifers 1-3
	Heifers > 3
	Cows 1-3
	Cows > 3
	Heifers 1-3 and cows 1-3
Housing dairy cows	Loose housing
	Tie stall
Housing heifers	Loose housing
	Tie stall
Number of cows going to communal alpine operation	1-5
	6-10
	11-15
	> 15

The questionnaire was pretested by four farmers not included in the study in order to assess the comprehensiveness of the questions.

Knowledge was assessed with specific statements to which the participants could answer with “true”, “false”, and “don’t know”. The attitude, behaviour and self-efficacy items were measured by a 6-point Likert’s scale [24] on how much they agreed or disagreed with the statement given (1 = completely agree, 6 = completely disagree). The preference for potential support during eradication was measured by multiple choice questions with the possibility to choose more than one of the suggested answers.

The questionnaire was mailed and a reminder was sent by mail 6 weeks later.

### Data analysis

Data analysis was performed with SPSS (SPSS Inc. Chicago IL, USA). A descriptive data analysis was performed, and results are given as means including standard deviations (SD) for normally distributed continuous data and frequencies were estimated for categorical data. The participants were divided into 2 groups based on their experience with a *S. aureus* herd problem (i.e. ≥ 1 positive cow in the herd within the past year). Group 1 were farmers who indicated that they never experienced a herd problem with *S. aureus* before and group 2 consisted of farmers, who indicated that they already had experienced a herd problem in the recent past.

The groups were compared using non-parametic tests i.e. χ^2^-test and Fishers exact test for frequencies ≤ 5. The siginficance level was set at *P* ≤ 0.05. The small sample size did not allow for logistic regression models.

## Results

A total of 90 questionnaires were sent out by mail and 43 were returned, resulting in a return rate of 48%. A total of 38 (42%) questionnaires were completed and were subjected to futher analysis.

### Description of sample

Among the respondents there was only one female (2.6%). In the following paragraph, the crude numbers and percentages of answers out of the total answers given to demographic items are listed. The majority of the respondents (16/31, 51.6%) operated farms of > 25 ha of surface, 32.2% (10/31) of 16-25 ha and 16.1% (5/31) of ≤ 15 ha. Twenty-one percent (8/37) of the respondents owned less than 10 cows, 37.8% (14/37) had 10-15 cows, 32.4% (12/37) had 16-25 cows and only 8.1% (3/37) owned > 25 cows. The main source of income was farming in 89.1% (33/37), and 54.1% (20/37) indicated that dairy production was the most important production area on their farm, whereas 24.1% (9/37) stated that veal calf production (using the whole milk of their dairy cows) was their main production area. The remaining respondents (8/37, 21.6%) chose different options such as veal and beef production, dairy and veal production on equal terms and egg production. Only 11% of the farmers produced according to the guidelines of BioSuisse (organic production). During winter, 47.2% (17/36) farmers used the milk of their dairy cows for the fattening of veal calves, 44.4% (16/36) sold it to a dairy factory and 8.3% (3/36) sold it to a raw milk cheese producer. Thirteen (36.1%) of 36 answers stated that the man power on their farms consisted of 2 persons with operating levels of 100% and 50%, respectively. Sixteen out of 36 farmers reported not having experienced a *S. aureus* problem (i.e. ≥ 1 positive cow in the herd, group 1) up to now, whereas 12 respondents indicated that they had dealt with a *S. aureus* problem during communal alpine pasturing in summer, 4 indicated that they had experienced a problem within the last 2 years, 1 respondent indicated that he currently dealt with a *S. aureus* problem and 2 respondents did not know if they ever have had a *S. aureus* problem in their herds.

### Attitude

#### General aspects of animal health

**Fig. 1:**
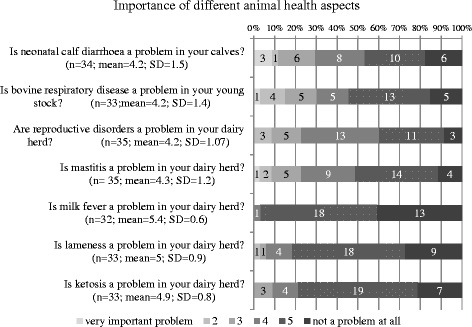
Distribution of self-reported importance of different cattle diseases in the study herds

The self-reported importance of different cattle health aspects with the mean and SD are displayed in Fig. 1. Mastitis seemed to be less important than reproductive disorders and calf diseases. Metabolic disorders such as milk-fever and ketosis were of much lower importance.

#### Attitude towards different udder-health aspects

**Fig. 2:**
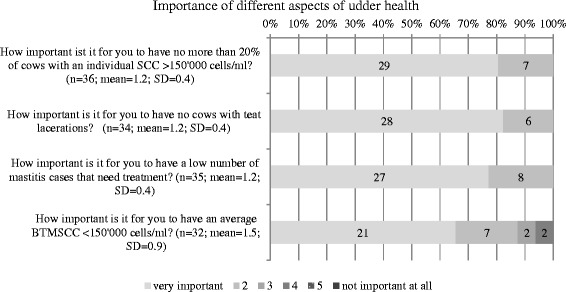
Distribution of self-reported importance of different aspects of udder health

**Fig. 3:**
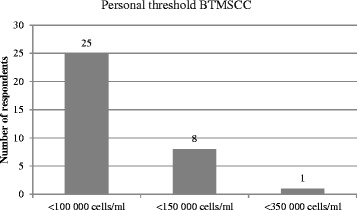
Frequencies of personal BTMSCC-threshold for satisfactory udder health

The distribution of the answers asking participants about self-reported importance of different udder health aspects with means and SD are shown in Fig. 2. The majority of the respondents recorded, that a bulk tank milk somatic cell count (BTMSCC) < 150,000 cells/ml, a low percentage of cows with elevated individual somatic cell counts, a low number of clinical mastitis needing treatment and a low number of teat lacerations were very important to them. Asked about a personal threshold of bulk tank somatic cell count considered as satisfactory, the majority claimed that they aimed at being < 100,000 cells/ml. The distribution of the answers is given in Fig. 3.

Twenty-nine participants out of 34 (85%) answering the questions stated that they would like to decrease their BTMSCC, only 5 out of 34 (15%) stated they dindn’t want to decrease the BTMSCC. Thirty-four out of 35 answers indicated, that the first person to seek advice in case of a mastitis problem in their dairy herd was their veterinarian. Only one respondent indicated to seek advice at the consultant for milking and milking equipment. The options “Swiss bovine health service”, “nutritionist”, “salesman for farm products” or “a colleague” were not chosen.

#### Attitude towards *S. aureus* control

The respondents were asked if they were willing to contribute to the control of *S. aureus* in the future. Twenty-three out of 34 (68%) respondents were willing to control *S. aureus*, 7 were not willing to participate in a control program and 4 chose the option “don’t know”.

**Fig. 4:**
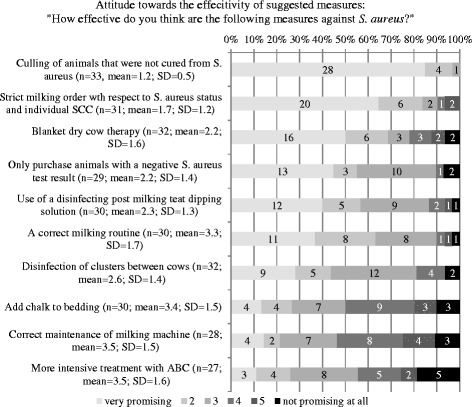
Distribution of answers to items on attitude towards effectiveness of generally suggested *S. aureus* control measures

Figure 4 shows the responses to questions about respondent’s attitude towards the effectiveness of different *S. aureus* control measures with means and SD. The measure “culling of infected animals with failure of treatment” was perceived as the most important measure followed by “sticking to a milking order according to the udder health status of the individual cows” and “milking according to a correct routine”.

#### Motivation

**Fig. 5:**
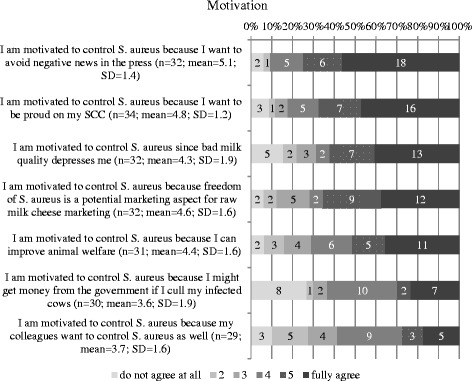
Distribution of factors motivating producers to participate in a *S. aureus* control program

**Fig. 6:**
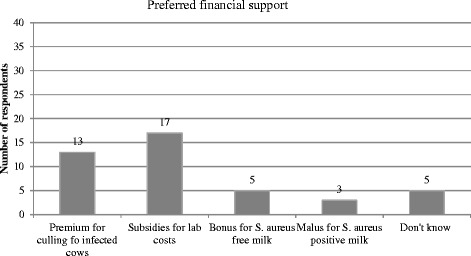
Frequencies of answers to the item about preferred financial support during a control program

Motivation was assessed with 7 items. “Avoiding negative news on raw milk cheese quality in the press” was one of the main motivators for respondents to control *S. aureus,* and they also saw some potential in the marketing of raw milk cheese free of *S. aureus*. Two slightly more emotional motivating factors were: “I want to be proud of my BTMSCC” (21/ 34) and “bad milk quality depresses me” (20/34). The distribution of the answers including the mean and SD is displayed in Fig. 5. The frequency of preferred financial support strategies i.e. external motivation factors is shown in Fig. 6, only one answer was possible. Seventeen out of 38 repondents preferred a discount on laboratory costs and 13 a premium for culling infected cows. A bonus for *S. aureus* free milk was preferred by 6 respondents, and only 3 thought a penalty system for *S. aureus* containing milk could serve as a motivating factor.

**Fig. 7:**
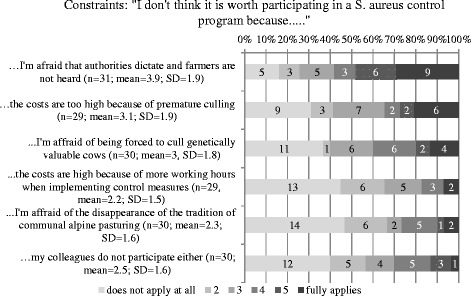
Distribution of constraining factors for participation in a *S. aureus* control program

The strongest constraint for participation in a *S. aureus* control program was the fear of an authority-dictated, top-down eradication program without taking into account the producers’ perspective. Distributions with means and SD are displayed in Fig. 7.

#### Preferred support in future control program

The most frequent answer to the question asking for preferred support in a potential future *S. aureus* control program was “regular support by the vet” (18 out of 35 answers), 8 of 34 respondents stated that no support was necessary, and 7 thought that a special education of personnel seasonally employed by communal alpine operations would be beneficial.

### Knowledge on *S. aureus*

**Fig. 8:**
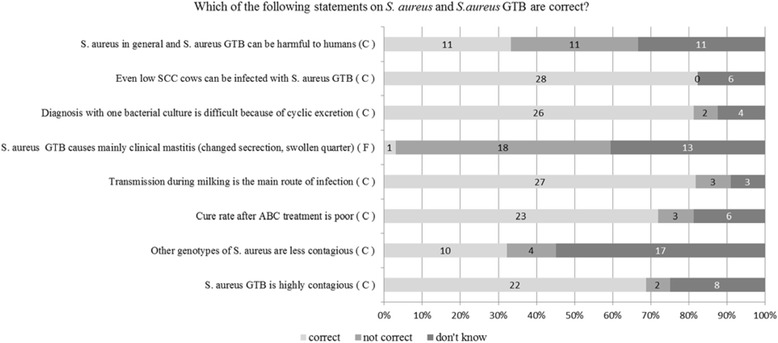
Frequencies of answers given by the farmers to items on knowledge (*S. aureus* and *S. aureus* GTB). The answers are marked either by C = scientifically correct or by F = scientifically incorrect, false

In Fig. 8, answers to specific questions about *S. aureus* and *S. aureus* GTB are displayed. The participants had to indicate, if they thought the statement was correct, not correct or if they did not know the answer.

### Behaviour

#### Mastitis management on cow level

**Fig. 9:**
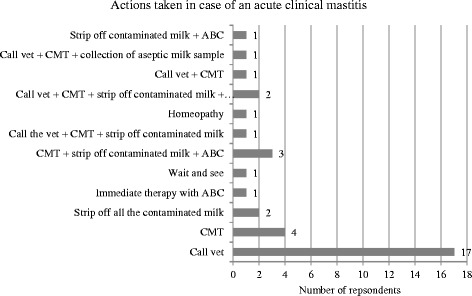
Frequencies of actions taken in case of acute clinical mastitis

The producers were asked which actions they take in case of an acute clinical mastitis. To avoid misinterpretation of terminology an example of a cow with a high body temperature, anorexia, swollen and painful quarter and changed milk secretion was given: “One of your cows is off feed, she has a body temperature of 41°C and her front right quarter is swollen and painful. What do you do first?” Frequencies of the different actions (options included: call vet, perform CMT, strip off contaminated milk, immediate treatment with antibiotics, homoeopathic treatment, wait and see, collection of milk sample) are shown in Fig. 9. Multiple answers were allowed.

**Fig. 10:**
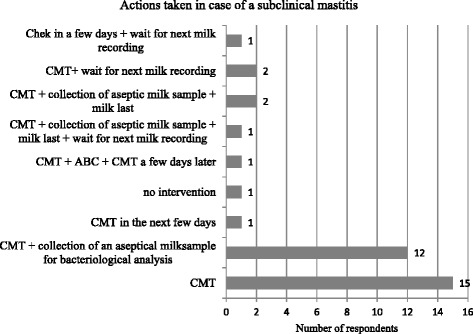
Frequencies of actions taken in case of subclinical mastitis

First actions taken in case of subclinical mastitis are displayed in Fig. 10 (options included: CMT immediately, CMT in the next few days, collection of aseptic milk sample, immediate treatment with antibiotics, homoeopathic treatment, milk last, wait for next test day result, no intervention). Again, to avoid misunderstanding of terminology an example of a typical case was given: cow without any systemic signs, elevated individual somatic cell count, positive CMT, no changes of quarter or milk secretion.

#### Monitoring udder health on herd level

**Fig. 11:**
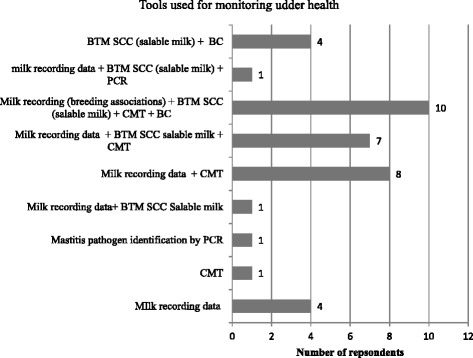
Frequencies of tools used to monitor udder health on herd level by the respondents

In Fig. 11 the frequencies of tools and combinations of different tools for monitoring of udder health given by 37 respondents are displayed. The farmers were asked “Which tools do you use to monitor udder health of your herd?”. The options included milk-recording data (individual somatic cell count, milk production), results of official BTMSCC measurements including weighed herd somatic cell count from milk recording and the BTM samples collected twice monthly by the milk company, CMT, bacterial culture (BC) and identification of mastitis pathogens by polymerase chain reaction (PCR). Multiple answers were allowed.

#### Belief in self-efficacy

**Fig. 12:**
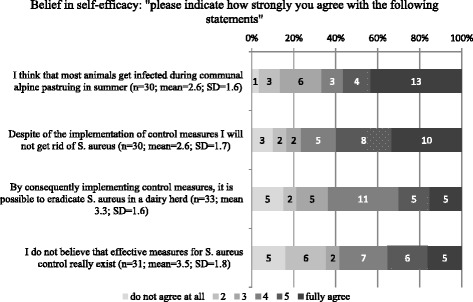
Distribution of answers concerning belief in self-efficacy

The answers to items asking participants about their trust in *S. aureus* control measures (means and SD) are displayed in Fig. 12. Although 21 of 32 respondents believed that consequent implementation of control measures will eradicate *S. aureus* from their herd, 18 of 30 respondents believed that they will never get rid of *S. aureus* despite implementation of control measures, and 11 of 31 respondents indicated that they do not believe in the existence of effective control measures at all. Out of 30 respondents, 13 were convinced that the majority of *S. aureus* infections occur during communal alpine pasturing in summer.

### Comparison of groups of respondents

Using χ^2^ tests to compare groups of farmers revealed that there were significant differences in terms of knowledge between farmers with a self reported experience of *S. aureus* within the previous year and farmers without such an experience. The knowledge items were recoded using categories according to the scientific correctness of an answer allocating 1 for scientifically correct answers and 0 for scientifically incorrect and “don’t know” answers. The number of scientifically correct answers to the statement “other *S. aureus* genotypes are less contagious than genotype B” was significantly higher in the group with an experience of a *S. aureus* problem (8 out of 13, 61.5%) than the number of correct answers given by farmers without an experience of a *S. aureus* problem (2 out of 18, 11.1%; *P* = 0.006). Farmers without a *S. aureus* experience gave significantly less scientifically correct answers (7 out of 18 38.9%) to the statement “*S. aureus* mainly causes clinical mastitis” and to the statement “Also low cell count cows can be *S. aureus* positive” (14 out of 20, 70%) than farmers with a *S. aureus* experience (11/14, 78.6%, *P* = 0.04 and 14 out of 14, 100%, *P* = 0.03, respectively).

The farmers who had experienced a *S. aureus* problem in the past year, had a tendency towards less rational constraints than farmers that had not been confronted with a *S. aureus* problem in the past year (Fisher’s exact *P* = 0.055).

## Discussion

This was the first study to assess attitude, knowledge and behaviour towards *S. aureus* control among raw milk cheese producing dairy farmers without a quota system.

Looking at the different attitude items, the respondents stated that mastitis was not the most important problem in their herds; reproductive failure and calf health seemed to be more important. This confirms results from a study conducted in the Netherlands stating that for most dairy farmers, mastitis is not first priority among herd problems they have to deal with [18].

Most participants indicated that a BTMSCC below 150,000 cells/ml was very important to them, this might be because some milk processers have established a bonus system for milk with a cell count ≤ 100,000 cells/ml [25]. The two respondents who indicated that a low BTMSCC was not important to them, were farmers, that used the milk of their cows exclusively for veal calf production during winter and, therefore, were not eligible to penalty rules of the milk companies. Although percentage of high SCC cows and BTMSCC are strongly linked, none of the respondents chose an option below 4 on the Likert scale when asked about the importance of a low percentage of high SCC cows in their herd. We can only speculate on the reasons, but almost all dairy farmers of the study including the veal producing farms were affiliated with a breeding organisation, and low SCC on the cows performance records are favourable for selling the animals.

Asked about the personal threshold of BTMSCC, the vast majority indicated a limit of 100,000 cells/ml, which was probably again influenced by the bonus system established in some milk companies. Additionally, it cannot be excluded that the answers given were at least partially socially desired. However, there was variation in the level of the personal threshold, confirming results of Kuiper et al. (2005) [15].

The majority of respondents (66%) were willing to improve the *S. aureus* situation, but 21% indicated that they saw no necessity in setting up a control program and 12% did not know. The strongest motivation factors to participate in a potential future *S. aureus* control program were: “avoiding negative press concerning cheese quality”, “*S. aureus* freedom of the milk might be a strong marketing argument for cheese sales” , “I want to be proud of my SCC” and “bad milk quality depresses me”. The strongest factors are not purely economically motivated, and confirm work that was done on lameness control in cattle where the strongest motivator was “I want to be proud of my herd” [26]. Valeeva et al., [19] reported as a major motivating factors to improve udder health “job satisfaction” and “overall situation of the farm” which is in line with the results of the present study. When asked about preferred financial support strategies, which can be interpreted as external motivation factors, a high number of participants chose “discount on laboratory costs” indicating that they were willing to invest in diagnosis of the causative agent of an udder health problem.

The major constraints for participation in *S. aureus* control programs reported by the respondents included: “I’m affraid that we will be forced into a top–down control program by the authorities without asking producers”, “economic loss because of premature culling of infected cows” and “lack of time to implement all the measures”. The factor “lack of time” was also found by others, asking about barriers to conrol lameness in dairy cattle [26]. The most frequent constraining factor identified in the current study should be taken into account when developing future disease control programs. It indicates that an integrative approach might be beneficial for the compliance with a control program, since the attitude of the farmers seems to be a key element in changing behaviour as described by others [18, 23]. “Culling of infected cows”, “no purchase of infected cows” and “milking infected cows last” were indicated as the three most useful measures to control *S. aureus,* but a large percentage did not believe in their ability to reach the desired outcome i.e. to control *S. aureus* by implementing control measures (“I believe that I will not get rid of *S. aureus* despite implementation of measures” and “I do not believe that effective measures to control *S. aureus* exist”). This might be associated with the low cure rate of *S. aureus* mastitis during lactation [11] and with the context of communal alpine pasturing, where comingling of animals from different farms of origin acts as a recurrent risk factor for infection with *S. aureus* every summer [27]. In a study investigating perceptions, circumstances and motivators to control zoonotic disease in cattle [21], a lack of belief in self-efficacy was associated with farmers without intention to control disease. Since we did not ask if specific herd-level control measures were implemented in order to assess intention to control, we cannot confirm the results of Ellis-Iversen et al. (2010) [21].

The knowledge concerning some key elements (such as contagiousness of different genotypes and clinical manisfestation) of *S. aureus* control differed significantly between the group of farmers with an experience of *S. aureus* herd problem compared to the farmers that reported no experience with *S. aureus* herd problem, although they all had attended an information event prior to the first mailing of the questionnaire. This difference might be explained by the fact that farmers, who dealt with a mastitis problem due to *S. aureus* in the past were actively looking for information. We can only speculate on that matter, because we did not include any questions on information sources. However, a recent Dutch study found, that increased knowledge on milking procedures and milking equipment was associated with decreased incidence of clinical mastitis [23]. It also indicated, that owners of affected herds were increasing their knowledge by consulting information sources or seeking advice by their veterinarians [23]. Ellis-Iversen et al. (2010) [21] found in their study that one of the most important extrinsic barriers for lack of disease control was lack of accessible knowledge. Unfortunately, we did not assess this in our study, but in the interviews conducted prior to the mailing of the questionnaire (data not shown), 2 out 4 farmers did not know what pathogens were involved in the mastitis cases occuring in their herds, because veterinarians only communicated how to treat the individual case but neither the result of bacteriological analysis nor how to proceed on herd level. This offers opportunities for udder health consulting by veterinarians as reviewed by Jansen & Lam (2012) [28]. Veterinarians were also reported to be the first persons, farmers would seek advice in case of an udder health problem confirming results of former studies [29] and emphasizing the important role of well educated practicioners in consulting and translation of knowledge.

Looking at the items about self-reported behaviour of mastitis management, the majority of respondents indicated to apply the recommended procedures in the management of mastitis cases, but no information on implemented herd-level measures were collected.

The present study is based on self-reporting and, therefore, socially desired answers cannot be excluded. Additionally, the data set of the present study was limited to very few respondents allowing only descriptive analysis and non-parametric tests for comparison of group 1 (experience with *S. aureus* problem) and group 2 (no experience with a *S. aureus* problem). Therefore, more sophisticated statistical analyses such as logistic regression models were not applicable and the present results should be confirmed on a larger scale. Nevertheless, our results on attitude and knowledge correspond well with other studies. The perception of farmers producing raw milk cheese under subsidised conditions without a quota system towards improving udder health by controlling *S. aureus* seem to be similar to perceptions of those producing under more industrial conditions.

## Conlcusions

The results of this study indicate that although 66% of farmers stated that they were willing to participate in *S. aureus* control, they were mainly constrained by the fear of an authority-dictated program without taking into account their individual situation. Therefore, it is crucial to integrate producers in the development of a regional control program. There is a need for harmonized knowledge transfer by veterinarians in order to fill knowledge gaps and to improve intention to control *S. aureus* mastitis.

## Additional file

Additional file 1: English translation of questionnaire. (PDF 120 kb)
