# Supplementary material for: Control of Staphylococcus aureus in dairy herds in a region with raw milk cheese production: farmers’ attitudes, knowledge, behaviour and belief in self-efficacy
Source: BMC Vet Res. 2018 Feb 13;14:46. doi: 10.1186/s12917-018-1352-0 (PMC5810121; doi:10.1186/s12917-018-1352-0)
Supplement: Supplementary file 1 — English translation of questionnaire. (PDF 120 kb) [file 12917_2018_1352_MOESM1_ESM.pdf]

Questionnaire: Udder health and udder infection with *Staphylococcus aureus*

Dear farmers

With the following questionnaire we would like to investigate your personal attitude, knowledege, practice and belief in self efficacy towards udder health problems including infections with *S. aureus*. Your statements may help to design a future control program for *S. aureus*. Experience from other countries (the Netherlands, UK) could show, that it is crucial to include the aspect of the animal owner when designing control program for animal diseases with an effect on the long term. That is why we need your information.

All given information will handled strictly confidential and data will used in an anonymized fashion exclusively for scientific purposes. Information will be used in case of the need of a regional *S. aureus* control program.

Thank you for your help!

Responsible for the project

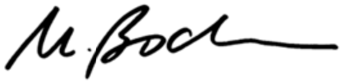

Michèle Bodmer, Dr. med. vet. dip. ECBHM, Clinic for Ruminants, University of Bern

Contact data of farmer

Name:

Forename:

Adresse:

Tel. No:

E-Mail:

---

1. General farm data

1.1. How many hectares of surface does your farm include?

1.2. In which cadastral zone is your farm situated?

Valley zone	☐
Prealpine zone	☐
Mountainous zone I	☐
Mountainous zone II	☐
Mountainous zone III	☐
Mountainous zone IV	☐

1.3. In which cadastral zone is your communal alpine operation?

Valley zone	☐
Prealpine zone	☐
Mountainous zone I	☐
Mountainous zone II	☐
Mountainous zone III	☐
Mountainous zone IV	☐

1.4. What is your herd size?

Cows: ______________________________

Relacement heifers: ______________________________

calves: ______________________________

1.5. Mode of operation in your farm?

Farm is main income	☐
Farm is 50% of income	☐
Farm is more than 60% of income	☐

1.6. Which main line of production in your farm?

____

1.7. How many persons are working on your farm? How many percent?

Only one person	☐	……………………………………………..%
1st Person	☐	……………………………………………..%
2nd Person	☐	……………………………………………..%
3rd Person	☐	……………………………………………..%
4th Person	☐	……………………………………………..%

1.8. How is your milk processed in winter??

Used to fatten calves	☐
Industrial milk	☐
Raw milk cheese production	☐

1.9. Do you produce according to regulation of a specific label during summer?

No label	☐
IP-Suisse®	☐
Bio Suisse®	☐
Terra Suisse®	☐
Coop Naturafarm®	☐

1.10. Do you produce according to regulation of a specific label during winter?

No label	☐
IP-Suisse®	☐
Bio Suisse®	☐
Terra Suisse®	☐
Coop Naturafarm®	☐

1.11. What is your replacement strategy?

Only own heifers raised on another farm	☐
Only own heifers raised on the home farm	☐
Own heifers raised on another farm and purchase	☐
Own heifers raised at home and purchase	☐
Only purchase, no own heifers	☐

1.12. How many lactating cows and heifers did you purchase in the past 12 months

Purchase of heifers: I__I__I heifers from I__I__I farms
Purchase of cows: I__I__I cows from I__I__I farms

2. Housing of cattle

2.1. Housing system of lactating cows?

Tie stall	☐
Loose housing system	☐

2.1.a) In case cows are kept in a tie stall, how the bedding on the lying surface?

Conventional rubber mats	☐
Comfort rubber mats	☐
Long straw	☐
Deep straw bedding	☐
Deep straw and chalk bedding	☐

2.1.b) In case you cows are kept in a loose housing system how is the bedding on the lying surface?

Cubicles with deep straw bedding	☐
Cubicles with deep straw and chalk bedding	☐
Cubicles with conventional rubber mats	☐
Cubicles with comfort rubber mats	☐
Deep straw bedding without cubicles	☐

2.2. How do you keep your replacement heifers older than 14 months?

Tie stall	☐
Loose housing	☐

3. Communal alpine pasturing

3.1. Name of the alpine operation you send your cows to?

___

3.2. How many of your lactating cows are brought to this communal operation?

___

3.3. From how many farms of origin do the cows derive?

___

3.4. How are the lying surfaces of the housing on the communal operation?

Conventional rubber mats	☐
Comfort rubber mats	☐
Wood	☐

4. Animal health and udder health

4.1. Which areas of animal health are worrying you currently and to what extent?

	Huge problem 1	2	3	4	5	No problem at all 6
Fertility	☐	☐	☐	☐	☐	☐
Mastitis	☐	☐	☐	☐	☐	☐
Claw health	☐	☐	☐	☐	☐	☐
Milkfever	☐	☐	☐	☐	☐	☐
Acetonaemia	☐	☐	☐	☐	☐	☐
Respiratory disease in young animals	☐	☐	☐	☐	☐	☐
Diarrhea in young animals	☐	☐	☐	☐	☐	☐

4.2. How important are following aspects of udder health for you?

	Very important 1	2	3	4	5	Not important at all 6
Bulk milk Somatic cell count below 150 000 Zellen/ml	☐	☐	☐	☐	☐	☐
A low number of mastitis cases that need treatment	☐	☐	☐	☐	☐	☐
Less than 20% of cows have high cell counts	☐	☐	☐	☐	☐	☐
A low number of teat lacerations	☐	☐	☐	☐	☐	☐

4.3. Which of the following tools do you use to monitor the udder health of your herd? (Multiple answers are possible!)

Data from regular milk recording by breeding federations	☐
Bulk milk cell count of the sold milk	☐
CMT	☐
Bacteriological analysis of milk samples	☐
Rotuine PCR identification of mastitis pathogens	☐
I do not use any tools	☐

4.4. One of you cows does not eat in the morning and she's got a fever (41°C) and here right hind quarter is hard and painful. What do you do <u>first</u> in this case??

I call the vet	☐
I perform a CMT	☐
I milk the cow immediately	☐
I milk her last (after the rest of the herd)	☐
I collect an aseptic milk sample	☐
I immediately treat her with antibiotics	☐
I wait and see	☐

4.5. One of your cows had an individual somatic cell count of 580‘000 cell/ml in the latest milk recording (increased from 80’000 cells/ml). There are no changes in any of the quarters and the milk looks normal. What do you do <u>first</u> in this case?

I perform a CMT	☐
I perform a CMT and collect an aseptic milk sample	☐
I milk the cow after the rest of the herd	☐
I immediately treat her with antibiotics	☐
I wait for the next milk recording	☐
I check the CMT over several days	☐
I wait and see	☐

4.6. Who do you adress first in case of an udder health problem?

The milking consultant	☐
The vet	☐
The bovine health service	☐
Colleagues	☐
The milking machine technician	☐
The salesman who sells the teat dipping product	☐
The feeding advisor	☐

4.7. At which cell-count threshold are you satisfied with the udder health of your herd?

If bulk tank somatic cell count iss maller than "_________".000 cells/ml

4.8. At which threshold you start thinking that there might be problem with the udder health in your herd?

If the bulk tank somatic cell count is above "_________".000 cells/ml

4.9. What was the actual mean in of the bulk tank somatic cell count in the last year?

"_________".000 cells/ml.

4.10. Would you like to decrease bulk tank somatic cell count?

Yes	☐
No	☐

4.11. If you had unlimited resources which of the following measures would you implement straight away to improve general udder health of your herd?

	Immediate implemen-tation				No implemen-tation	
	1	2	3	4	5	6
No purchase of animals	☐	☐	☐	☐	☐	☐
Purchase only animals with negative lab result	☐	☐	☐	☐	☐	☐
No communal alpine pasturing	☐	☐	☐	☐	☐	☐
Changes in housing	☐	☐	☐	☐	☐	☐
Improve hygiene	☐	☐	☐	☐	☐	☐
Improve hygiene at milking	☐	☐	☐	☐	☐	☐
Improve milking routine	☐	☐	☐	☐	☐	☐
Introduce a milking order accroding to udder health status oft he individual cows	☐	☐	☐	☐	☐	☐
New milking machine	☐	☐	☐	☐	☐	☐
More frequent maintenance of milking machine	☐	☐	☐	☐	☐	☐
Change feeding of cows	☐	☐	☐	☐	☐	☐
Change dry off management	☐	☐	☐	☐	☐	☐
Monitoring of udder health with vet (herd health visits)	☐	☐	☐	☐	☐	☐
Cull problem animals	☐	☐	☐	☐	☐	☐
Hire more people to improve animal management	☐	☐	☐	☐	☐	☐

4.12. If you imagine having a serious udder health problem in you dairy herd. What are the barriers that keep you from implementing measures?

	Important constraint 1	2	3	4	5	Not important at all 6
My financial situation	☐	☐	☐	☐	☐	☐
I do not have an udder health problem in my herd	☐	☐	☐	☐	☐	☐
Lack of time	☐	☐	☐	☐	☐	☐
There is no successor taking over my farm and I don’t want to make any changes anymore	☐	☐	☐	☐	☐	☐
I will stop dairy production in 1 to 2 years	☐	☐	☐	☐	☐	☐
The work routine is disturbed by the measures	☐	☐	☐	☐	☐	☐
Because of a lack of infrastructure it is not possible for me to implement mastitis control measures	☐	☐	☐	☐	☐	☐
Don’t know	☐	☐	☐	☐	☐	☐

4.13. Which pathogens were isolated in you herd in the past 2 years? (Multiple answers possible!)

E. coli	☐
Streptococcus uberis	☐
Streptococcus spp.	☐
Staphylococcus spp.	☐
Staphylococcus aureus	☐
C. bovis	☐
Don’t know	☐

5. Subject Staphylococcus aureus (*S. aureus*)

5.1. Were you already confronted with a S. aureus problem in your herd?

There is no S. aureus problem in my herd at the moment	☐
I currently have a serious S. aureus problem in my herd	☐
I had a serious S. aureus problem 1 year ago	☐
I had a serious S. aureus problem several years ago	☐
Yes, during communal pasturing in summer	☐
Don’t know	☐

5.2. If you experienced a S. aureus problem how long was its duration?

3 moths	☐
6 months	☐
1 year	☐
More than 1 year	☐
More than 1 year and is still present	☐
Don’t know	☐

5.3. Which statements concerning S. aureus genotype B* (GTB) are correct

	True	False	Don't know
S. aureus GTB highly contagious	☐	☐	☐
Other types of S. aureus might be less contagious	☐	☐	☐
Cure rates after antibiotic treatment are poor	☐	☐	☐
The main route of transmission is during milking	☐	☐	☐
S. aureus GTB causes mainly clinical mastitis (changed milk, hard quarter)	☐	☐	☐
S. aureus GTB mainly lives in the mammary gland but is not always shed and is therefore not always found in milk cultures	☐	☐	☐
Cows with low somatic cell counts (<100 000 cells/ml) can be S. aureus GTB positive	☐	☐	☐
S. aureus can be harmful to humans	☐	☐	☐

* is the genotype that was frequently found in the region, the farmers were all informed about this issues

5.4. Imagine you got a serious problem with S. aureus GTB in your dairy herd. Which of the following measures do you think are effective to control S. aureus?

	Very promising 1	2	3	4	5	Not promising at all 6
Purchase of negative animals	☐	☐	☐	☐	☐	☐
Culling of not cured animals	☐	☐	☐	☐	☐	☐
Very intensive antibiotic treatment	☐	☐	☐	☐	☐	☐
Dry off with antibiotics	☐	☐	☐	☐	☐	☐
Strict milking order (1st Healthy animals; 2nd suspicous animals 3rd infected animals)	☐	☐	☐	☐	☐	☐
Desinfection of milking clusters between cows	☐	☐	☐	☐	☐	☐
Correct milking routine and hygiene	☐	☐	☐	☐	☐	☐
Add chalk to the bedding	☐	☐	☐	☐	☐	☐
Maintenance of milking machine	☐	☐	☐	☐	☐	☐
Post milking teat disinfection with effective product	☐	☐	☐	☐	☐	☐

5.5. Please indicate how strongly you agree with the following statements on the effectiveness of S. aureus control.

	Fully agree 1	2	3	4	5	Don't agree at all 6
I strongly believe that I can control S. aureus with the strict implementation of measures within 1 year.	☐	☐	☐	☐	☐	☐
I believe, that despite implementing control measures I'll always have to live with S. aureus	☐	☐	☐	☐	☐	☐
I don't believe that effective measures to control S. aureus really exist.	☐	☐	☐	☐	☐	☐
I believe that the infection mainly takes place during communal alpine pasturing.	☐	☐	☐	☐	☐	☐
Don't know	☐	☐	☐	☐	☐	☐

5.6. Which oft he following statements on S. aureus GTB are correct?

	True	False	Don't know
S. aureus GTB is a huge problem for the regional dairy production	☐	☐	☐
S. aureus GTB is only a problem for the affected herds	☐	☐	☐
S. aureus GTB is only a sporadic disease	☐	☐	☐
S. aureus GTB is a problem in ma herd	☐	☐	☐
S. aureus GTB impaires the taste of our cheese	☐	☐	☐
S. aureus GTB impaires the quantity of the produced cheese	☐	☐	☐
S. aureus GTB impaires the soundness for human consumption of our cheese	☐	☐	☐
S. aureus GTB does not have negative effects on human health	☐	☐	☐
S. aureus GTB-toxins can cause cause food poisoning in humans	☐	☐	☐
Only immune-compromised humans can get sick from S. aureus GTB	☐	☐	☐
If we use a lot of antibiotics fot S. aureus treatment we might as well suffer from resistances ourselves.	☐	☐	☐

6. Improvement of the S. aureus situation

6.1. Do you think that an initiative to control S. aureus should be launched?

Yes	☐
No	☐
Don't know	☐

6.2. I'm motavated to participate in a potential control program because…

	Fully agree 1	2	3	4	5	Do not agree at all 6
…bad milk quality depresses me.	☐	☐	☐	☐	☐	☐
…I would like to be proud of my cell count.	☐	☐	☐	☐	☐	☐
…my colleagues want to participate as well.	☐	☐	☐	☐	☐	☐
…I feel that S. aureus control is also a good marketing tool to sell our cheese	☐	☐	☐	☐	☐	☐
…I want to prevent negative press	☐	☐	☐	☐	☐	☐
…I might get a premium when I slaughter infected animals	☐	☐	☐	☐	☐	☐
…I can improve animal welfare	☐	☐	☐	☐	☐	☐

6.3. I don't want to participate in a S. aureus control program because….

	Fully agree 1	2	3	4	5	Don't agree at all 6
…I dont have a S. aureus problem.	☐	☐	☐	☐	☐	☐
…we never had any problems with cheese quality.	☐	☐	☐	☐	☐	☐
…I cannot implement the control measures because I don't have the time	☐	☐	☐	☐	☐	☐
…my colleagues don't want to participate	☐	☐	☐	☐	☐	☐
…the measures will cost too much because of additional work	☐	☐	☐	☐	☐	☐
…the measures will cost too much because of premature culling of animals	☐	☐	☐	☐	☐	☐
…I fear that the authorities will force me to cull valuable cows because they are positive	☐	☐	☐	☐	☐	☐
…I fear that the tradition of communal alpine pasturing and cheese production will disappear	☐	☐	☐	☐	☐	☐
…I fear that the communal alpine pasturing will be prohibited and I will lose a lot of money because there is no cheese sale anymore	☐	☐	☐	☐	☐	☐
…I fear that the authorities dictated a program and the producers are not asked	☐	☐	☐	☐	☐	☐

6.4 What kind of support would you prefer?

Support by vet	☐
Regular support by milking consultant	☐
Special education for personnel on communal operations	☐
Regular meetings of farmers in discussion groups	☐
Don’t know	☐

6.5. Who should have the responsibility for a control program?

The authorities	☐
Vet	☐
Producers	☐
Milkprcessors	☐
Milking consultants	☐
Behörden + Produzenten	☐
Authoriteis + producers + vets	☐
Authoriteis + producers + vets + milking consultants	☐
Authoriteis + producers + vets + milking consultants + milkprocessors	☐
Don’t know	☐

6.6. Which of the following options concerning financial support would you prefer?

Premium for culling positive cows	☐
Subsidies for milk analysis	☐
Bonus for S. aureus free milk	☐
Malus for S. aureus positive milk	☐
Don’t know	☐

Thank you for completing this questionnaire!
